# The Deep Learning–Based Recommender System “Pubmender” for Choosing a Biomedical Publication Venue: Development and Validation Study

**DOI:** 10.2196/12957

**Published:** 2019-05-24

**Authors:** Xiaoyue Feng, Hao Zhang, Yijie Ren, Penghui Shang, Yi Zhu, Yanchun Liang, Renchu Guan, Dong Xu

**Affiliations:** 1 Key Laboratory of Symbolic Computation and Knowledge Engineering of the Ministry of Education College of Computer Science and Technology Jilin University Changchun China; 2 Department of Electric Engineering and Computer Science University of Missouri Columbia, MO United States; 3 Zhuhai Sub Laboratory, Key Laboratory of Symbolic Computation and Knowledge Engineering of the Ministry of Education Zhuhai College Jilin University Zhuhai China; 4 Christopher S Bond Life Sciences Center University of Missouri Columbia, MO United States

**Keywords:** recommender system, deep learning, convolutional neural network, biomedical literature, PubMed

## Abstract

**Background:**

It is of great importance for researchers to publish research results in high-quality journals. However, it is often challenging to choose the most suitable publication venue, given the exponential growth of journals and conferences. Although recommender systems have achieved success in promoting movies, music, and products, very few studies have explored recommendation of publication venues, especially for biomedical research. No recommender system exists that can specifically recommend journals in PubMed, the largest collection of biomedical literature.

**Objective:**

We aimed to propose a publication recommender system, named Pubmender, to suggest suitable PubMed journals based on a paper’s abstract.

**Methods:**

In Pubmender, pretrained word2vec was first used to construct the start-up feature space. Subsequently, a deep convolutional neural network was constructed to achieve a high-level representation of abstracts, and a fully connected softmax model was adopted to recommend the best journals.

**Results:**

We collected 880,165 papers from 1130 journals in PubMed Central and extracted abstracts from these papers as an empirical dataset. We compared different recommendation models such as Cavnar-Trenkle on the Microsoft Academic Search (MAS) engine, a collaborative filtering–based recommender system for the digital library of the Association for Computing Machinery (ACM) and CiteSeer. We found the accuracy of our system for the top 10 recommendations to be 87.0%, 22.9%, and 196.0% higher than that of MAS, ACM, and CiteSeer, respectively. In addition, we compared our system with Journal Finder and Journal Suggester, which are tools of Elsevier and Springer, respectively, that help authors find suitable journals in their series. The results revealed that the accuracy of our system was 329% higher than that of Journal Finder and 406% higher than that of Journal Suggester for the top 10 recommendations. Our web service is freely available at https://www.keaml.cn:8081/.

**Conclusions:**

Our deep learning–based recommender system can suggest an appropriate journal list to help biomedical scientists and clinicians choose suitable venues for their papers.

## Introduction

### Background

With the fast-growing research activities, more biomedical papers are being published in thousands of journals worldwide. For example, PubMed Central (PMC) has 5.2 million papers and 7409 journals covering biomedical and life sciences [1]. Although these publications play a major role in disseminating research outcome, the growth of journal publications imposes a challenge for selections of appropriate publication venues. It is vital that authors submit to the right journal that meets the journal scope and provides sound reviews. It is equally important that they reach their intended audience and obtain a large number of citations [2]. However, researchers are unfamiliar with all the journals related to their work for choosing the most suitable one for submitting a paper. Moreover, different publication scopes of journals and research interests of reviewers and editors may affect the decision of a submitted manuscript. If the submitted paper cannot meet the interests of a publication venue and its editors and reviewers, it may lead to rejection, delay, or less readership. An appropriate recommender system can help solve this problem.

Recommender systems have been proven to serve as an effective method for decision making in many areas such as music, movies, and information media choices [3-6]. The well-known techniques of recommender systems are content-based recommendation [7,8], collaborative filtering recommendation [4,9], and hybrid recommendation [6,10]. Content-based recommender systems recommend an item to a user based on a description of the item. Collaborative filtering methods and hybrid methods may outperform the content-based recommendations by applying user data, if available. However, after the user privacy issue of Facebook in 2018 and the introduction of European Union General Data Protection Regulation, user data are no longer easy to obtain. Moreover, in many domains, especially in material recommendation, there are no user data available for collaborative filtering methods at the beginning [11], which is regarded as a cold-start problem. Content-based recommendations do not need any user information and are more suitable for solving these problems [12].

Based on the content-based recommendation strategy, several attempts have been made to create recommender systems for medical applications and scientific literature. Using geotagged mobile search logs, Agarwal et al [13] adopted a Random Forest model to predict medical visits. Using topic, writing style, author information, citation information, abstract, and title as information items, latent Dirichlet allocation [14] and k-nearest neighbor [15] were used to classify the scientific literature for recommendation [2,12,16,17]. Luong et al [18] used the coauthors’ network as advanced information to recommend a publication venue. Beel et al [19] conducted a literature survey on recommender systems, exploring their methods, evaluation measurements, and datasets. For most of these recommender systems, the high-dimensional and sparse matrix computation is a critical problem [20].

Because of the mismatches caused by ambiguity in text comparisons, the content-based recommendation approach may cause a high error rate [21]. Recently, due to the ability of discovering intricate structures and deep semantics in high-dimensional data, deep learning methods have succeeded in many areas and recently been proposed to build recommender systems for both collaborative filtering and content-based approaches. Hinton et al [22] proposed restricted Boltzmann machines for modeling tabular or count data as a collaborative filtering model on the Netflix data set. McAuley et al [23] proposed an image-based recommendation, which adopted a deep learning model to extract image features. Van den Oord et al [24] applied a deep convolutional neural network (CNN) to predict latent factors from music audios for music recommendation. Wang et al [11] proposed a collaborative deep learning model to jointly perform deep representation learning for the content information and collaborative filtering of a rating matrix. However, to the best of our knowledge, these deep learning techniques have not been used in any biomedical literature recommender system.

Most current venue recommendation studies focus on computer science and technology, but not on the biomedical field. Biomedical sciences are highly interdisciplinary and often link to engineering, medicine, biology, physics, psychology, etc, thereby serving more journals and more diverse topics than any other field. Hence, the development of a recommender system is more essential and challenging for the biomedical sciences than any other discipline. Furthermore, previous recommender systems were based on shallow machine learning methods and social networks. They were generally keyword-based methods and did not take semantics into account. In addition, the few existing systems only focus on journals under a certain organization, such as Elsevier, IEEE, and Springer, instead of PubMed.

### Aim

In contrast to our previous study on computer science publication recommendations using conventional machine learning approaches [12], we proposed a deep learning–based recommender system for biomedical publication venues, named Pubmender. Due to the copious vocabulary of biomedical literature, the traditional vector space model can lead to high-dimensional and sparse problems. To address this issue, dimensionality reduction methods are needed before learning the pattern. Moreover, initializing text matrix by pretrained word embedding is more beneficial for training neural networks than random initialized embedding [25]. Accordingly, we applied a word2vec model for our study instead of using the conventional vector space model employed in our previous publication venue recommender system. In addition, deep learning models are able to learn multiple-level abstract representations of data with syntactic and semantic information, since more abstract concepts can be constructed with multiple processing layers [26]. We applied the deep learning approach to provide recommendations of journals for biomedical researchers. Unlike shallow learning, the state-of-the-art embedding method and deep CNN in Pubmender were trained from 837,882 papers in 1130 biomedical journals. This method can help researchers find a variety of choices, without being limited to their own knowledge of journals.

## Methods

### Pubmender System

**Figure 1:**
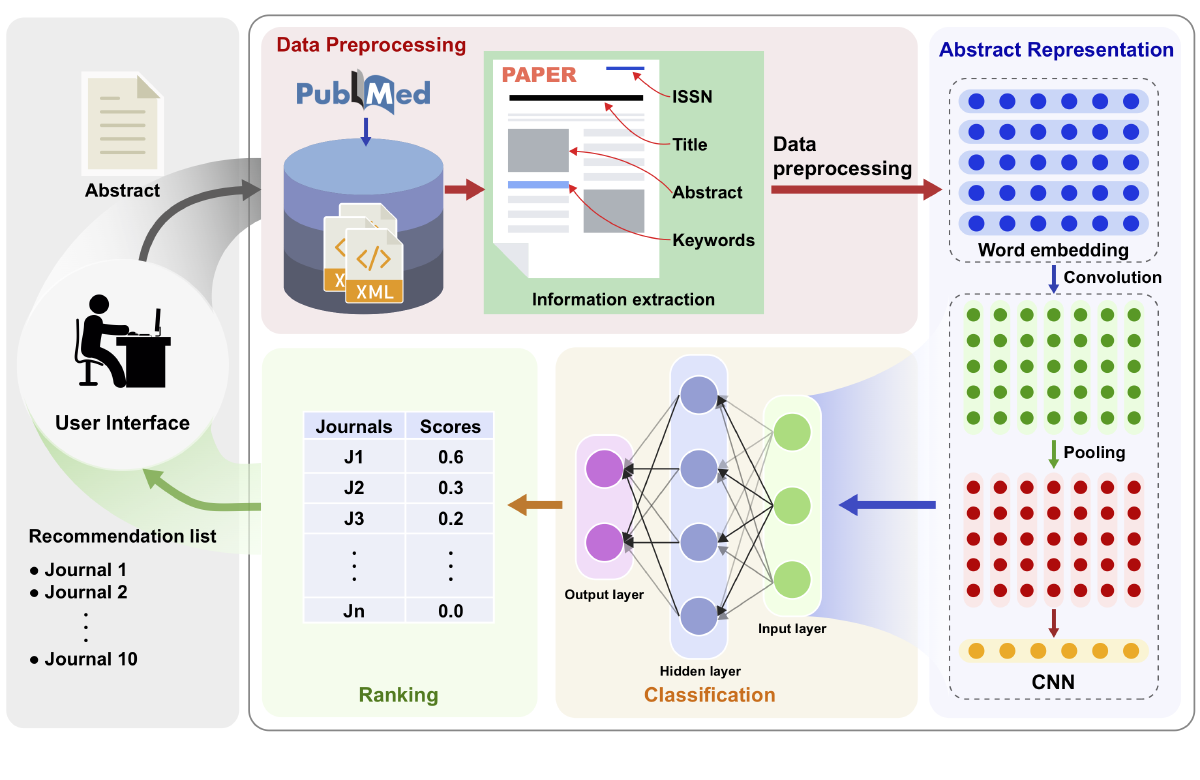
Architecture of our Pubmender system. CNN: convolutional neural network; ISSN: International Standard Serial Number.

Figure 1 shows the architecture and workflow of our Pubmender system. It consists of user interface, data preprocessing, abstract representation, classification, and ranking phase.

The user interface obtains the input data (an abstract submitted by a user) and presents the recommendation results to the user. The data acquirement is followed by data preprocessing and information extraction. At the start of our deep learning model, the abstract representation phase converts an abstract to a vector. The original abstract vector is a concatenation of pretrained word vectors. Subsequently, deep CNN is applied to train the model to achieve high-level abstract representation. A three-layer fully connected network with a softmax operation is applied to classify papers based on the obtained abstract vectors. The recommendation list of the top N journals obtained from the ranking phase is presented to the user.

### Data Preprocessing Methods

The data were downloaded from the File Transfer Protocol service of PubMed Central (PMC) [27], containing 1,534,649 papers. Based on the journal list of PMC, we selected normal journals deposited under full participation or the US National Institutes of Health portfolio mode, excluding records labeled “Predecessor,” “No New Content,” and “Now Select.” Papers from Jan 2007 to Apr 2017 were selected. Papers with no abstracts or with fewer than 200 characters in abstracts were deleted. Journals containing fewer than 100 papers were also removed. Finally, 880,165 papers in the XML format from 1130 journals were used in our study.

Each PMC file is a semistructured XML document and contains various tags, such as <title>, <abstract>, and <issn>. We extracted the content in <abstract>, <ISSN>, and <pub-date> fields from the raw XML files. Then, pissn and eissn in the ISSN field were replaced by “LocatorPlus ID,” which is the unique identification for a journal in the US National Library of Medicine catalog. After extraction, each abstract was stored in a corresponding file. Natural Language Toolkit was adopted to operate word segmentation [28].

### Abstract Representation

In Pubmender, the recommendation task is formulated into a multilabel classification problem, where the text representation and classification methods are critical. For abstracts, we originally embedded abstracts with pretrained word vectors. Thereafter, the original embeddings were fed into CNN to achieve more abstract representation as explained below.

Let **v**_*i*_∈R^*k*^ be the *k*-dimensional word vector corresponding to the *i*-th word in the abstract *A*. An original representation of *A* is represented as a matrix **V**={**v**_1_,…,**v**_*m*_}^T^, which is the concatenation of the words’ vectors. Due to the different sizes of abstracts, we set *m* as the maximum count of words in an abstract. A padding operation with zeros was adopted for input with fewer than *m* words in an abstract and a tail truncation operation for more than *m* words. The vectors of words adopt pretrained vectors using word embedding and are induced from the PubMed abstracts and PubMed Central full text. The word2vec tool [29] was adopted for word embedding using the skip-gram model with a window size of *h*, hierarchical softmax training, and a frequent word subsampling threshold 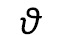
to create *k*-dimensional vectors. Word vectors are initialized by zeros if they are not in the pretrained vocabulary. Finally, the representation of an abstract is matrix **V** with a dimensionality of m*k. It was used as the input to feed to the next step. To achieve more abstract and semantic features, we adopted CNN to extract semantic information.

**Figure 2:**
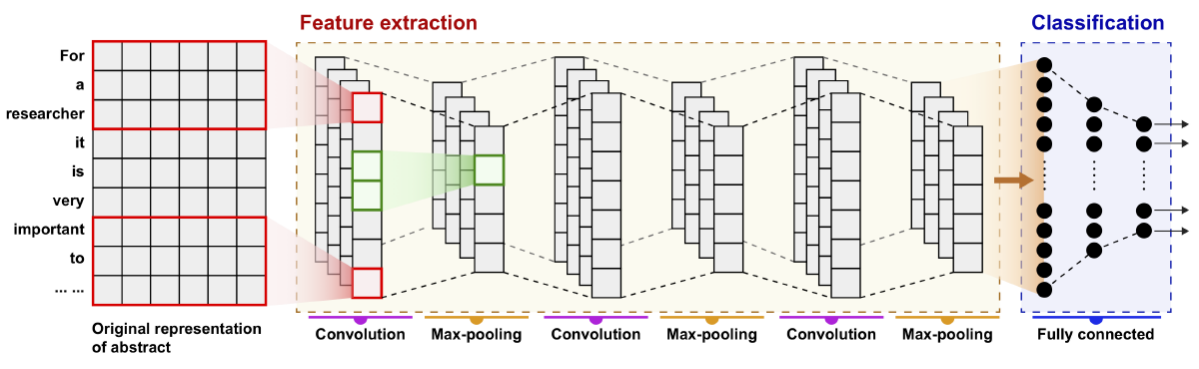
The structure of our deep convolutional neural network model.

Figure 2 shows the structure of our deep CNN model. There are three convolutional and max-pooling layers in CNN, one fully connected layer, one hidden layer, and one softmax layer for classification. For an abstract, *A*(*w*_1_,*w*_2_,...,*w_n_*) with *w_i_* represents the *i-*th word and **v**_*i*_∈R^*k*^ is the *k*-dimensional word vector corresponding to word *w_i_*. The abstract is represented as **v**_1:*m*_=**v**_1_⨁**v**_2_⨁...⨁**v**_*m*_ (1), where ⨁ is the concatenation operator, *m* is the maximum length of abstracts (a scalar), and **v***_i:i__+__j_* refers to the vector of concatenation of the words *w_i_*,*w_i_*_+1_,…,*w_i_*_+__*j*_. The first convolutional layer performs as a one-dimensional convolution operation on sliding windows of *h*_1_ words to produce a phrase feature. For example, a feature *c_ji_* is generated from a window of words **v**_*i:i+h*_1_–1_ by *c_ji_*=*g(f_j_*▪**v**_*i:i+h*_1_–1_+**b**_1_) (2). Here, **b**_1_∈R is a bias term and *g* is a nonlinear function such as rectified linear unit (ReLu). *f_j_*∈R^*k* × *h*_1_^ is the *j*-th convolutional kernel, whose shape is *k* × *h*_1_, where *k* is the dimension of word vectors and *h*_1_ is the window size. This kernel is applied to each possible window of words in the abstract {**v**_1:*h*_1__,**v**_2:*h*_1_+1_,...,**v**_*m–h*_1_+1:*m*_} to produce a feature map **C**_*j*_=[*c*_*j*1_, *c*_*j*2_...,*c*_*j,m–h*_1_+1_] (3) with **C**_*j*_∈R^*m–h*_1_+1^.

If there are *r*_1_ convolutional kernels, then 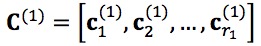
is the result of the first convolution operation on **V**. The pooling operation is then carried out on **C^(1)^**. Its function is to progressively reduce the spatial size of the representation to extract the key features and reduce the number of dimensions in the network. The pooling layer operates independently on every depth slice of the input and resizes it spatially, using the max-pooling operation [30] in every two-unit window for each **C_j_^(1)^**. **P^(1)^**, described below, is the result of the max-pooling operation: 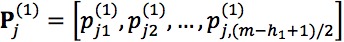 (4), where *j* is the *j*-th filter of the convolutional operation 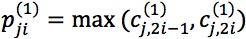 (5).

The second and third convolutional and pooling layers work the same as Equations (2) and (5). Following the three convolutional and pooling operations is the fully connected layer. Here, the input is represented with a more abstract feature 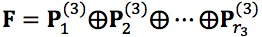, where *r*_3_ is the number of third-layer convolutional filters. The three convolutional and pooling operations indicate a phrase-level feature, a sentence-level feature, and an abstract-level feature. The algorithm of abstract embedding is listed in Textbox 1.

**Abstract embedding algorithm.**

**Input:**

- Embedding the abstract *A* to matrix **V**={**v**_1_,…,**v***_m_*}^T^
- *r_t_* is the number of convolutional filters of layer *t*, where *t*=1, 2, 3
- *h_t_* is the convolutional window size of layer *t*, where *t*=1, 2, 3

**Output:**

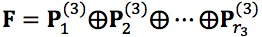

**Procedure:**

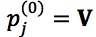

2. for *t*=1, 2, 3
3. for *j*=1, 2,…*r_t_*
4. for *i*=1, 2,…*m–h*_t_+1

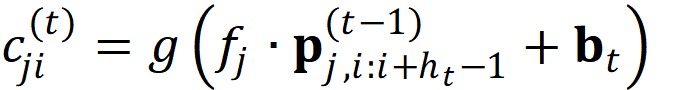

6. End for
7. for *i*=1, 2,… (*m*–*h_t_*+1)/2

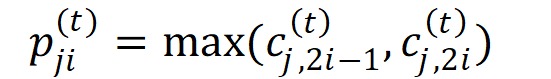

9. End for

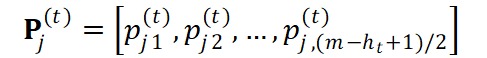

11. End for

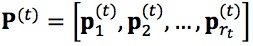

13. End for

### Softmax Classification

A fully connected softmax layer is the last layer of Pubmender. Given the training sample, *A*, where *T* is the number of possible labels, *z* is the class score for the sample, and the estimated probabilities *S_j_*∈[0,1) for each label j∈{1,2…*T*} the softmax formula is: 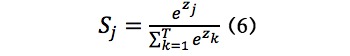


We trained the entire model by minimizing the cross-entropy error defined as 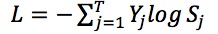
(7), where *Y* is the true classification output. This is a one-hot encoding of size *T*, where all elements except one are 0, and one element is 1. This element marks the correct class for the data classified. We employed the optimizer Adam to learn the model parameters, which is a variant of stochastic gradient descent [31].

## Results

### Datasets

After data preprocessing, there were 880,165 preprocessed papers from PMC in 1130 open-access journals from Jan 2007 to Apr 2017. The “LocatorPlus ID” assigned to each journal by PMC is regarded as the classification label of a paper. We generated four data sets based on these papers. The first data set included all papers from 2007 to 2016, which was used to choose the feature representation method and train the prediction models. Papers in 2017 formed the second data set, which was used as the test set to verify Pubmender’s performance. The last two datasets chose papers from publications in Elsevier and Springer from 2017, which were used to compare our Pubmender with Journal Finder and Suggester. The statistics of the first dataset are described in Table 1.

One of the biggest challenges of these datasets is that the data distribution is highly imbalanced. In the first dataset, 60 journals published more than 2000 papers, while 740 journals published fewer than 400 papers. The number of papers in “PLOS One” was 153,608, which is larger than the number in other journals, based on its extensive and comprehensive scope. “Scientific Reports” ranked second, with 37,864 papers published, while “Horticulture Research” only published 100 papers. The average paper count was 741, and 934 journals had fewer than that number.

**Table 1:** Details of the first dataset (Jan 2007 to Dec 2016).

Statistic		Number of journals^a^	Number of papers^b^
**Size**			
	100≤x^c^≤400	740	157,038
	400<x≤2000	330	259,676
	2000<x≤10,000	55	195,426
	>10,000	5	225,742
	Total	1130	837,882
Maximum class size		1	153,608
Minimum class size		4	100
Average class size		N/A^d^	741

^a^This represents the total number of journals in this range.

^b^This represents the total number of papers published in all journals in this range.

^c^x represents the number of papers published in one journal.

^d^N/A: not applicable.

### Parameters and Measurements

For CNN, three convolutional and three pooling operations were adopted. The pretrained word vectors generated by word2vec, available from Evex [29], were used. The window size *h* was 5 and threshold 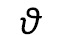
was 0.001. The dimension of a pretrained vector was 200, that is, *k*=200. The length of abstract had a fixed size *m*, which is the maximum number of the words that most abstracts contain. Table 2 shows the word statistic details of the papers. With the statistics, only 43,328 of 837,882 papers (5%) contained abstracts with more than 350 words. Therefore, we chose *m*=350. A zero-padding operation was applied for abstracts with fewer than 350 words, together with a tail-truncation operation for abstracts containing more than 350 words.

The convolutional operation parameters are listed in Table 3. The activation function adopted the ReLU function. In the pooling layer, the size of max-pooling filters was two, applied with a stride of two down samples. The parameters of the following layers (pooling layers) had the same parameter settings. Normalization and dropout strategies were used in the fully connected layer. Rate of dropout was 0.2 and L_2_ normalization was adopted.

### Evaluation of Recommendation Results

#### Toy Experiment

We designed a toy experiment to validate the deep learning method. In the first dataset, 421,168 papers were chosen from 60 journals with more than 2000 papers. The training set and test set contained 37,951 (90%) and 4,217 (10%) papers, respectively. We selected bi-directional long short-term memory (Bi-LSTM) and fastText [32] as comparison models for Pubmender. Bi-LSTM represents the recurrent neural network model with the max-pooling operation from a previous study [33]. Pretrained word vectors, generated by word2vec from a previous study [29], were used as the input original word vectors for fastText and Pubmender.

To evaluate the performance of our system, top-*N* accuracy was adopted as a measurement, which is defined as the probability that the expected label is in the top *N* predicted classes. For top-*N*, if the journal containing the abstract is among the top *N* ranked journals, the classification is correct. The symbol acc@*N* represents the accuracy of top-*N*, *N*=1, *N=*3, and *N*=5. The comparison of accuracy is listed in Table 4, which shows that both deep learning approaches outperformed fastText in all the three measurements. The accuracy of Bi-LSTM is nearly the same as that of Pubmender. However, the running time of Pubmender was 2660 abstracts per second, which is 78% faster than Bi-LSTM (1495 abstracts/second), and Bi-LSTM needs more memory.

**Table 2:** Word statistics of abstracts.

Size	Number of abstracts
20≤x^a^<50	25,499
50≤x<100	76,614
100≤x<150	139,420
150≤x<200	227,993
200≤x<250	191,156
250≤x<300	87,597
300≤x<350	46,275
x>350	43,328

^a^x denotes the number of words in the abstract.

**Table 3:** Hyperparameters of convolutional operation.

Convolutional layer	Convolution kernel count	Window size
First	256	3
Second	128	4
Third	96	5

**Table 4:** Accuracy of Bi-LSTM, fastText, and Pubmender. Italicized values indicate the best results. acc@N represents the accuracy for top-N selection.

Methods	acc@1	acc@3	acc@5
fastText	0.66	0.86	0.92
Bi-LSTM^a^ (max-pooling)	0.71	0.90	0.95
Pubmender	*0.72*	*0.92*	*0.96*

^a^Bi-LSTM**:** bi-directional long short-term memory.

#### First Dataset Result

For the first dataset, there were 837,882 papers in 1130 journals from 2007 to 2016. The training, validation, and test sets contained 670,306 (80%), 83,788 (10%), and 83,788 (10%) randomly selected papers, respectively. The results of and comparisons with previous work are provided in Table 5.

The other two systems were from three widely used digital libraries: Association for Computing Machinery (ACM) [16], CiteSeer [16], and Microsoft Academic Search (MAS) [2]. The results from Table 5 show that Pubmender achieved the best performance. The proposed system can achieve 0.50 on acc@1 and 0.86 on acc@10. Our system improved performance by 225% over CiteSeer and MAS in terms of acc@5, and by 87% over MAS and 196% over CiteSeer in terms of acc@10. The system described by Yang and Davidson [16] used topic and writing-style information, and the system described by Medvet et al [2] used the abstracts and titles. However, our Pubmender obtained the best accuracy by using abstracts only.

To present the ability of handling imbalanced data of Pubmender, we divided the test set into four classes (tiny, small, medium, and large) according to the paper counts of different journals. From Table 6, for the tiny set, Pubmender achieved 0.27 accuracy on the acc@1 and 0.54 on acc@5, which are greater than the accuracy on acc@5 and acc@10 (in Table 5) from MAS and CiteSeer, respectively. The accuracy of the top-10 (acc@10) of a large set reached 0.98. In the paper by Medvet et al [2], 58,466 papers were partitioned almost uniformly into 300 conferences from the MAS. In CiteSeer [16], 35,020 selected papers were published across 739 venues, each of which had at least 20 papers. The average number of papers for each venue was 47. Therefore, the CiteSeer dataset is almost balanced. In contrast, the imbalance of our data, as shown in Table 1, was very critical. The sizes of classes in our dataset ranged from 100 to 153,608 papers; for example, the number of papers in “PLOS One” was 153,608, which is 270 times the average number of papers in all journals. Compared with balanced data, the classification of critically imbalanced data was a complex problem to tackle. For this problem, our model achieved satisfactory results.

**Table 5:** Accuracy of the classification by Pubmender and other systems. Italicized values indicate the best results. acc@N represents the accuracy for top-N selection.

Methods	Paper count	Journal count	acc@1	acc@3	acc@5	acc@10
Pubmender	837,882	1130	*0.50*	*0.71*	*0.78*	*0.86*
MAS^a^ [2]	58,466	300	—^b^	—	0.24	0.46
ACM^c^ [16]	172,890	2197	—	—	0.56	0.70
CiteSeer [16]	35,020	739	—	—	0.24	0.29

^a^MAS: Microsoft Academic Search.

^b^Experimental evaluation is not available.

^c^ACM: Association for Computing Machinery.

**Table 6:** Pubmender accuracy at top N(@N) of imbalance class data. acc@N represents the accuracy for top-N selection.

Paper count range	acc@1	acc@3	acc@5	acc@10	Paper count
Tiny	0.27	0.44	0.54	0.66	16,337
Small	0.43	0.63	0.72	0.82	26,259
medium	0.62	0.81	0.88	0.94	19,588
Large	0.66	0.91	0.96	0.98	22,579
All	0.50	0.71	0.78	0.86	84,763

Moreover, excluding accuracy, we choose precision, recall, and the F1-score as measurements. For an individual class **C**_*i*_, the assessment is defined by *tp_i_* (true positives), *fp_i_* (false positives), *fn_i_* (false negatives), and *tn_i_* (true negatives). Accuracy, precision, and recall are calculated from the counts for **C**_*i*_. Quality of the overall classification is evaluated in two ways: macro-averaging and micro-averaging. The macro-average is the average of the same measures calculated for all classes. With the sum of counts to obtain cumulative *tp*, *fp*, *tn*, and *fn*, micro-average metrics are calculated [34]. The following equations show how the desired results are individually achieved: 
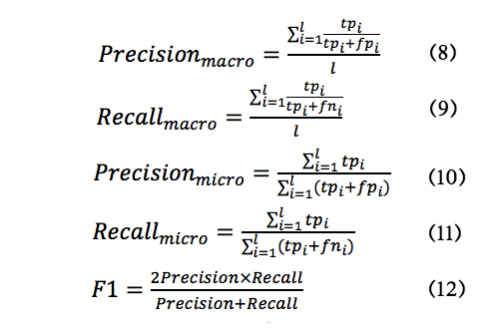


We listed macro-average and micro-average metrics in Table 7. Macro-averaging treats all classes equally, while micro-averaging favors bigger classes. From Table 7, it can be seen that when the number of recommended journals increases, the probability of capturing the real journal also increases. Therefore, the recall is increased step by step from top-1 to top-10. With the growth in the number of recommended journals, the number of falsely selected journals is also growing, which results in a decrease in precision. The F1-score favors a balanced view.

#### New Data Verification

To show the performance on new data, 42,283 papers from January 2017 to April 2017 were extracted to make further predictions. This comprised 1321 journals, some of which did not appear in the first dataset. These unseen journals increased the difficulty of prediction. The accuracy of Pubmender on the top-1, 3, 5, and 10 was 0.39, 0.61, 0.68, and 0.76, respectively. The accuracies on acc@5 and acc@10 were 183% and 162% higher than those of CiteSeer, respectively. From these results, we conclude that our proposed recommender system achieves a satisfactory result, even for new data that may not belong to the same data distribution.

### Comparison with Journal Finder and Journal Suggester

Journal Finder is provided by Elsevier for recommending Elsevier journals [35]. There are 45 Elsevier journals in our dataset. Five of them with more papers were selected. The paper counts were 582 for *Medicine*, 193 for *Data in Brief*, 124 for *NeuroImage: Clinical,* 117 for *Redox Biology*, and 87 for *Preventive Medicine Reports*.

Elsevier’s Journal Finder requires input of the title and abstract of submitted paper, and fields of research. The titles and abstracts were extracted from XML files and then fed into Journal Finder. We chose fields of research in “Engineering,” “GeoSciences,” “Life and Health Science,” and “Chemistry.” The results are listed in Table 8 and show that our system is much better than Journal Finder.

**Table 7:** Macro-average and Micro-average metrics for recommendation results.

Metrics	Macro-average			Micro-average		
	Precision	Recall	F1	Precision	Recall	F1
Top-1	0.38	0.32	0.33	0.50	0.50	0.50
Top-3	0.37	0.50	0.41	0.45	0.71	0.55
Top-5	0.35	0.59	0.42	0.42	0.78	0.55
Top-10	0.32	0.70	0.42	0.38	0.86	0.53

**Table 8:** Comparison between Pubmender and Journal Finder. Italicized values indicate the best results. acc@N represents the accuracy for top-N selection.

Systems	acc@1	acc@3	acc@5	acc@10
Pubmender	*0.62*	*0.75*	*0.84*	*0.90*
Journal Finder	0.05	0.12	0.13	0.21
Improvement (%)	1140	525	546	329

**Table 9:** Comparison between Pubmender and Journal Suggester. Italicized values indicate the best results. acc@N represents the accuracy for top-N selection.

Systems	acc@1	acc@3	acc@5	acc@10
Pubmender	*0.57*	*0.81*	*0.87*	*0.91*
Journal Suggester	0.11	0.15	0.17	0.18
Improvement (%)	418	440	412	406

Journal Suggester [36], recommends journals published by Springer. Journal Suggester also requires input of the title and abstract, and field of research. We chose “Biomedicine” as the field of research. There are 14 journals from Springer in our dataset, and seven of them were chosen for comparison based on a significant number of papers in each journal — *Cell Death & Disease*, *Malaria Journal*, *Nanoscale Research Letters*, *Nature Communications*, *Parasites & Vectors*, *Scientific Reports*, and *Trials*. Each journal chose the top 100 papers according to the size of the XML files. The results are listed in Table 9. Again, our system was much better than Journal Suggester.

## Discussion

In this study, Pubmender was proposed to recommend a biomedical publishing venue to user. CNN was used to obtain the abstract representation. Our results show the performance of the system.

### Principal Results

For biomedical publications, our Pubmender system is the first recommender system with word embedding and deep learning models. It achieves 87.0%, 22.9%, and 196.0% higher accuracy than recommender systems on MAS, ACM, and CiteSeer, respectively. In addition, the experiment results also revealed that the accuracy of our system was superior to that of Journal Finder and Journal Suggester. Our web service is freely available online [37].

### Comparison with Prior Work

Because no paper has been published about biomedical venue recommendations, we cannot perform any exact comparison with previous work. However, some publishers provide tools to help authors choose suitable journals. We chose two tools provided by Elsevier and Springer for comparison. The first one is Journal Finder provided by Elsevier for recommending journals of Elsevier. Pubmender achieved a much higher accuracy than Journal Finder on four metrics. For example, on acc@1, the accuracy of our system reached 0.62 and Journal Finder was given an accuracy rating of 0.05, with 1140% improvement; on acc@10, the accuracy of our system reached 0.84, which is 546% higher than that of Journal Finder. Pubmender also significantly outperformed another tool, Journal Suggester.

### Conclusions

In this study, we proposed a biomedical publishing venue recommender system—Pubmender. In this system, an abstract is first represented by a vector using the composition of pretrained word vectors. Subsequently, a deep CNN architecture is designed to represent and classify the submitted abstract. The original vectors are converted into more abstract feature vectors containing semantic information using deep CNNs, which overcome the sparse high-dimensional problem.

The experimental results showed that our proposed system achieves more successful performance than that of MAS, ACM, CiteSeer, Journal Finder, and Journal Suggester. Even for journals containing a small number of abstracts, the performance of Pubmender was satisfactory, because Pubmender’s high-level representation method catches more semantic and structural information from the abstract.

## Abbreviations

ACM: Association for Computing Machinery
Bi-LSTM: bi-directional long short-term memory
CNN: convolutional neural network
MAS: Microsoft Academic Search
PMC: PubMed Central
ReLu: rectified linear unit
